# A Case of Glossitis—Mal-treatment, Nearly Resulting in Death

**Published:** 1848-01

**Authors:** T. P. Bicknell


*A Case of Glossitis—Mal-treatment, nearly resulting in Death.* By T. P. Bicknell, M.D. E. W———th, a lad æt. 12, was attacked about two weeks since by this rare, although not to be mistaken disease. A quack was called in, who came to the wise conclusion that it was a "difficulty!!" in the mouth!!! and ordered poultices under the chin; saying, that he would draw it to a *head*, then he *could let the matter out, and thereby effect a cure!!*"

The child grew worse rapidly; the friends became alarmed, and in about six days from the commencement of the disease, I was called to see him. I found the tongue hot, red and swollen, filling the whole cavity of the mouth, and thrust out between the teeth, appearing like a mass of raw flesh. The respiration was extremely difficult, deglutition almost impossible. I immediately made two deep incisions into the substance of the tongue, from which issued nearly a pint of dark, grumous blood, with evident relief; prescribed nauseating does of vinum antimonii every hour, and left him quite comfortable. In about three hours the incisions closed and the tongue rapidly increased in size. The quack was again called, who persuaded the friends that I was killing the child. Another quack was sent for in order to hold a consultation! They agreed as to the "difficulty!" and sagely concluded that there was matter under the chin, "which must *be let out.*" They then proceeded to puncture under the chin, and of course found no "matter;" but told the friends there would be plenty of "matter" in a few days.

The child grew worse, and was momentarily in danger of suffocation. The father came to me about twelve hours after the puncture under the chin had been made, and with tears in his eyes implored me to take charge of the patient, saying that he was satisfied the child must die under their course of treatment.

At this late period I again took charge of him. With a scalpel introduced flatways between the tongue and the teeth, I made two deep incisions nearly dividing the whole substance of the tongue from the root to the tip, from which again issued above a pint of dark offensive grumous blood, which afforded almost instantaneous relief. In twenty hours he could shut his teeth over his tongue, and articulate distinctly; on the third day he was nearly well, the incisions in his tongue healing kindly.—*Illinois and Indiana Med. and Surg. Jour.*

